# Fabricating robust thin film composite membranes reinforced on woven mesh backing fabric support for pressure assisted and forward osmosis: A dataset

**DOI:** 10.1016/j.dib.2018.10.007

**Published:** 2018-10-04

**Authors:** Soleyman Sahebi, Ho Kyong Shon, Sherub Phuntsho, Bahman Ramavandi

**Affiliations:** a Department for Management of Science and Technology Development, Ton Duc Thang University, Ho Chi Minh City, Vietnam; b Faculty of Environment and Labour Safety, Ton Duc Thang University, Ho Chi Minh City, Vietnam; c Centre for Technology in Water and Wastewater, School of Civil and Environmental Engineering, University of Technology Sydney (UTS), PO Box 123, 15 Broadway, NSW 2007, Australia; d Environmental Health Engineering Department, Faculty of Health and Nutrition, Bushehr University of Medical Sciences, Bushehr, Iran

**Keywords:** Thin film composite, Forward osmosis, Pressure assisted osmosis, Woven mesh backing fabric support

## Abstract

The data presented in this paper are produced as part of the original research article entitled “Thin-film composite membrane on a compacted woven backing fabric for pressure assisted osmosis” (Sahebi et al., 2017). This article describes how to fabricate a defect free membrane for forward osmosis (FO) and pressure assisted osmosis (PAO) on the woven mesh backing fabric support. Casting polymer on backing fabric support may limit the interfacial polyemirization due to wrinkled membrane surface. This paper presents data obtained from two different backing fabrics used as support for fabrication of thin film composite FO membrane. Backing fabric support were woven polyester mesh with different opening size. The data include the characterization of the intrinsic properties of the membrane samples, SEM and their performance under FO process. The structural parameters (S value) of the substrate were computed from thickness and porosity of the substrates. Thin film composite (TFC) membrane achieved a water flux of 8.1 L m^2^ h^−1^ in FO process and 37 L m^2^ h^−1^ using 0.5 M NaCl as draw solution (DS) and deionised (DI) water as the feed solution (FS) when applied hydraulic pressure was 10 bar.

**Specifications table**

**Table:** 

Subject area	Separation and purification technology
More specific subject area	Forward osmosis, pressure assisted osmosis
Type of data	Figure, table
How data was acquired	Ultrasonic cleaners (Soniclean Pty Ltd, Australia)
	Stainless steel film applicator (Sheen Instruments Ltd, UK)
	Schottky Field Emission Scanning Electron Microscope (SEM, Zeiss Supra 55VP, Carl Zies AG, Germany)
	Balzers Sputter coater (SCD 050, BAL-TEC, Germany) was operated at the 10–15 kV applied voltage.
	Optical Tensiometer (Attension Theta Lite 100, Biolin Scientific, Finland)
Data format	Analysed
Experimental factors	Prepared dope polymers were cast on the compacted woven backing fabric supports with the 5% open area and then interfacial polymerization was performed on the top surface of the substrates to form rejection polyamide layer and FO membranes were achieved.
Experimental features	The fabricated membranes were characterized and later assessed under forward and pressure assisted osmosis.
Data source location	University of Technology Sydney (UTS), Australia, Sydney
Data accessibility	With this article
Related research article	This data is presented as a companion paper to the original research article entitled: “Thin-film composite membrane on a compacted woven backing fabric for pressure assisted osmosis”, by S. Sahebi et al. published in the Journal of Desalination [1].

**Value of the data**

• The presented data is very useful for researchers who want to develop FO membranes on a woven or non-woven backing fabric support.
• This data provides a valuable data to troubleshoot the membrane fabrication on backing fabric support during phase inversion. Findings led to use of compacted fabric instead of conventional polyester mesh woven support fabric to limit the polymer penetration as a main cause of wrinkling on the membrane surface.
• The key parameters in applying fabric support that were involved on membrane top surface wrinkles and defects were revealed.

## 1 Data

**Fig. 1:**
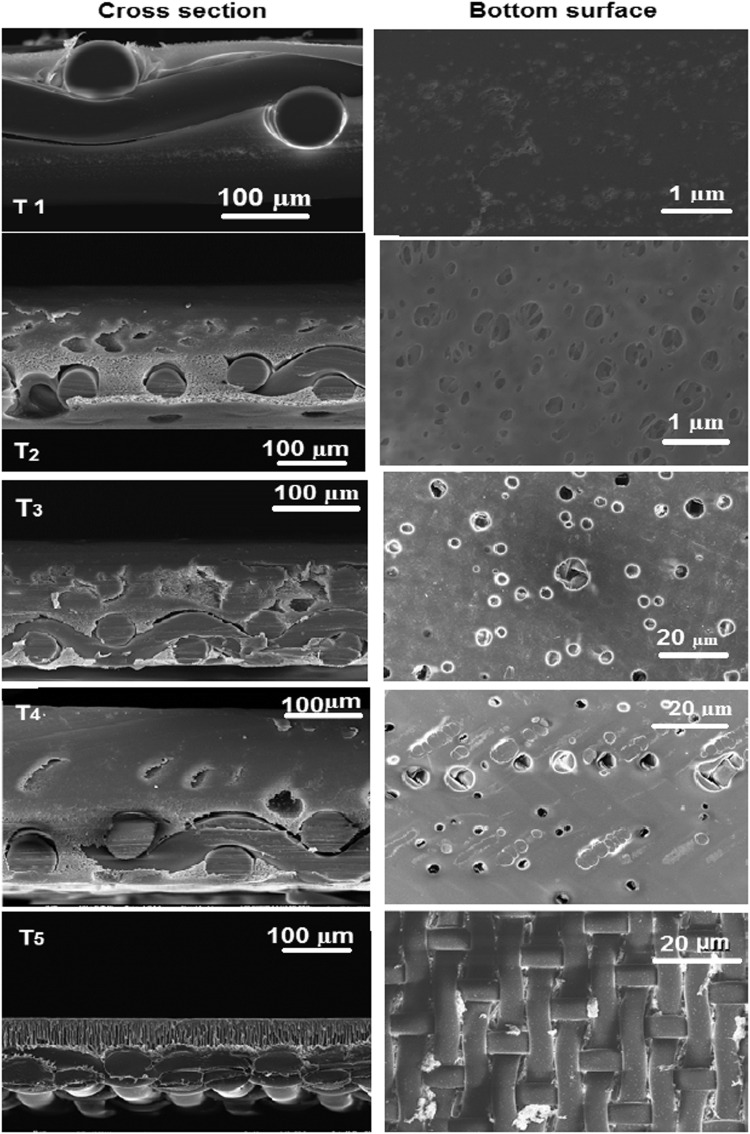
SEM images of cross-section and the bottom surfaces for the fabricated samples on woven mesh backing fabrics denoted as T_1_–T_5_ (Table 3 illustrates the fabrication methods, materials and composition for synthesis of all samples).

**Fig. 2:**
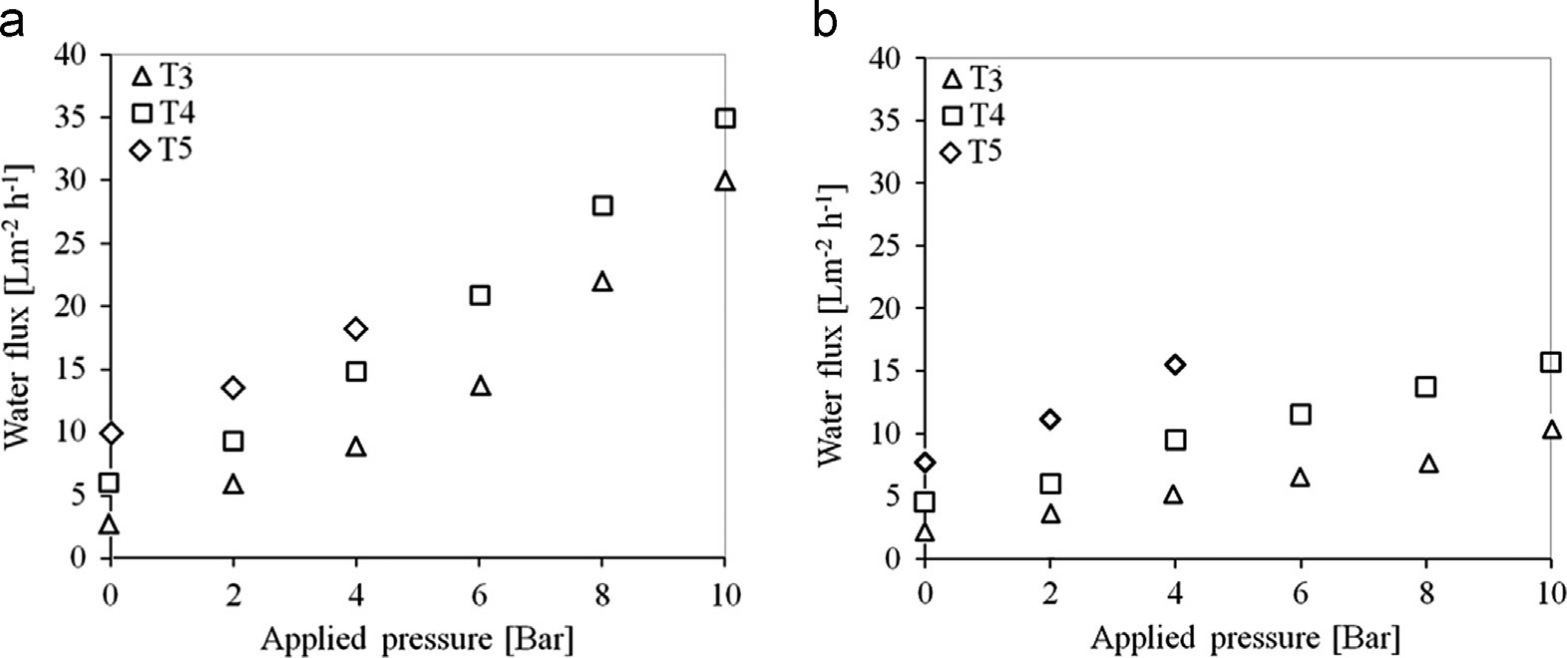
Performance of fabricated membrane samples in terms of water flux **(a)** DI water as FS and 0.5 M NaCl as DS and, **(b)** BW10 as FS and 0.5 M NaCl as DS at (0–10 bar) applied hydraulic pressure to assess PAO and FO processes (Note: T_5_ membrane sample could not stand applied hydraulic pressure more than 4 bar due to macrovoid morphology that is prone to compaction (Table 1, Fig. 1)).

**Fig. 3:**
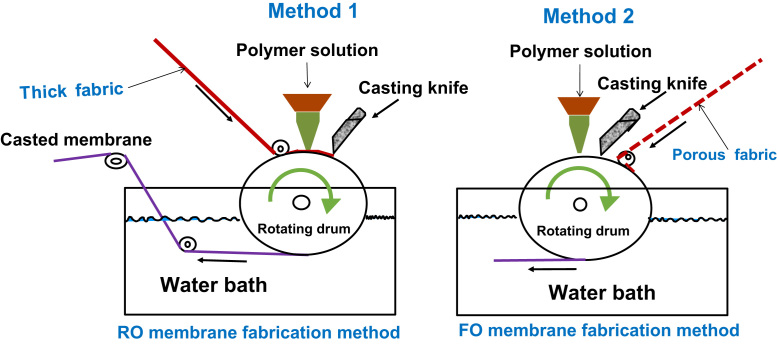
Schematic of RO and FO membrane fabrication methods in commercial scale [4].

Table 1, Table 2 present the characterizations and properties for the membrane samples. Table 3 show the polymer composition, back fabric and fabrication methods used for developing suitable FO membrane in this study. For the fabrication of flat-sheet TFC membranes for FO processes, two woven polyester mesh fabrics with different opening area (5% and 25%) were applied as backing fabric support. Fig. 1, Fig. 2 present the SEM images and performances in terms of water flux. Fig. 3 shows the schematic of RO and FO membrane fabrication methods in commercial scale.

**Table 1:** Characterisation of membrane substrates.

Membrane ID	Thickness (µm)		Porosity (%)	Contact angle (°)	
	Virgin	^*^Used		Active layer	Support layer
T_1_	99.3	–	71	54	74
T_2_	105.2	–	70	51	72
T_3_	150.2	142	72	56	75
T_4_	155.3	141	74	57	76
T_5_	103.2	72	79	55	75

**Table 2:** Properties of fabricated TFC membrane for FO processes (results were achieved for 3 samples as successful interfacial polymerization could not be performed in samples T_1_ and T_2_).

Sample ID	aPure water permeability L/m^2^ h^−1^ bar^−1^	bSalt permeability B (10^−8^ m/s)	NaCl rejection (%)	bSalt permeability B (10^−8^ m/s)	B/A (kPa)	FO Water flux (L m^−2^ h^−1^)	SRSF (g L^−1^)	*S* value (mm)
T_3_	2.2 ± 0.16	5.6 ± 0.13	96.2	5.6 ± 0.13	9.15	5.1	1.4	2.72±0.15
T_4_	3.2 ± 0.26	14 ± 0.13	94.3	14 ± 0.13	14.2	6.7	1.6	2.21±0.15
T_5_	3.3 ± 0.25	18 ± 0.13	91.2	18 ± 0.13	18.2	11	2.1	0.72±0.17

a Assessed in the RO testing mode over a different applied pressure range of 1–10 bar. DI water was used as feed water.

b Assessed in the RO testing mode over a different applied pressure range of 1–10 bar for a feed water with 1–10 g/l NaCl.

**Table 3:** Composition for the synthesis of the membrane samples fabricated in this work.

Sample ID	Fabric open area (%)	Composition polymer dopes			Fabrication method
		PES (wt %)	NMP (wt %)	PEG (wt %)	
T_1_	25	18	72	10	FO
T_2_	25	18	72	10	RO
T_3_	5	18	72	10	RO
T_4_	5	18	62	20	RO
T_5_	5	12	88	–	RO

## 2 Experimental design, materials, and methods

### 2.1 Casting membrane for FO and RO in a large scale

Fig. 3 demonstrates the two fabrication methods on the roll (rotating drum) used for the fabricating of the RO and FO membranes at a large scale. Method 1 (M_1_) is used for casting the RO membrane using a thick non-woven fabric support with highly viscose solution. In this method, the non-woven backing fabric is firstly pulled on the rotating drum and then the dope solution is flowed via nuzzle on the top of the thick nonwoven fabric and cast by a blade on top of the rotating drum [2], [3], whereas for fabrication FO membrane, the dope solution is first casted onto a rotating roll and then; the woven mesh fabric is dragged onto the polymer film from the top to fully embed the backing fabric [2].

A comprehensive explanation of the casting procedure for fabricating FO membranes supported by backing fabric on a commercial scale is described in the HTI patent [2] which is similar to the fabrication method 2 presented in Fig. 3. This method has been chosen in commercial scale in order to limit the polymer solution bleeding through the woven mesh fabric in the CTA-FO membrane which otherwise leads to defect points across the FO membrane substrate due to trapped air bubbles [1], [5]. In method 2, polymer solution first is casted on the rotating drum then the porous fabric will be pulled from the top whereas in RO style membrane fabrication (method 1) polymer solution directly is poured and casted on the nonwoven thick fabric.

### 2.2 Measurement of intrinsic properties of the membrane

Intrinsic properties of the TFC membranes were evaluated by RO testing mode. The *A* value (water permeability) was achieved based on the following equation:

(1) $$A=\frac{\Delta V_{a}}{\Delta t_{a}\times A_{m}\times \Delta P}$$

The *A* value was calculated by placing DI water in the feed container and applying hydraulic pressure of 5.0 bar. Δ_*Va*_ is the amount of permeate water over a time which is Δ*ta* and *Am* is the membrane area and Δ*P* is the applied pressure difference.

NaCl rejection was calculated based on the following the following equation.

(2) $$R=\frac{\mathrm{Cf}-\mathrm{Cp}}{\mathrm{Cf}}\times 100\%$$

where *C*_*f*_ and *C*_*p*_ are the salt concentrations in the feed and permeate, respectively.

The following equation was used for calculating salt permeability (*B*) of a membrane:

(4) $$B=\frac{A(1-R)(\Delta p-\Delta \pi )}{R}$$

where *R* is the salt rejection of the membrane, Δ*p* is the applied pressure and Δπ is the osmotic pressure difference for the semipermeable membrane.

### 2.3 Calculating membrane structural parameter

*S* value or membrane structural parameter is given by the following equation which can be calculated by achieving membrane tortuosity (τ), support layer thickness (t) and its porosity (*ε*):

(5) $$S=\frac{t\tau }{\varepsilon }$$

### 2.4 Membrane tests under FO and PAO processes

**Fig. 4:**
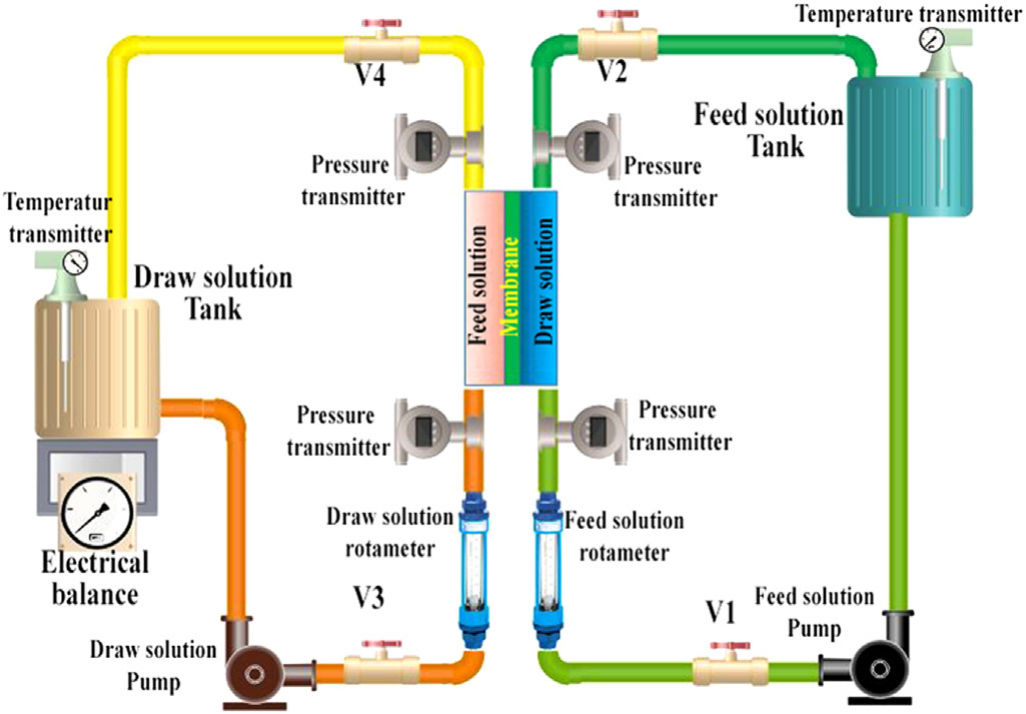
Schematic of the lab-scale experimental setup for pressure assisted osmosis process.

Membrane tests under the FO and PAO process were evaluated in FO cell presented in Fig. 4. All experiments are tested under FO mode of operation. Hydraulic pressure was applied on the FS side in the case of PAO tests. An adjustable pressure pump was used for the FS side [1], [4], [6], [7], [8]. The applied hydraulic pressure on the FS varies between 0 and 10 bars. The reverse solute flux (RSF) was measured by observing the electrical conductivity (EC) applying a multimeter (CP-500L, EK, Korea) where DI water is used as FS [7], [9].
